# Supplementary material for: Cultural variation in young children’s social motivation for peer collaboration and its relation to the ontogeny of Theory of Mind
Source: PLoS One. 2020 Nov 19;15(11):e0242071. doi: 10.1371/journal.pone.0242071 (PMC7676710; doi:10.1371/journal.pone.0242071)
Supplement: S1 File — (DOCX) [file pone.0242071.s001.docx]

**– Additional Analyses and Details of Analyses –**

**Details on Model 1**

**Step 1:**

**Comparing Full Model with a null model not comprising the predictors**

***Model Comparison***

|  | **Chisq** | **df** | ***p*** |
| --- | --- | --- | --- |
| Full Model | 96.32 | 14 | < .001 |

**Step 2:**

**Model 1 – Full Model comprising 2-way interactions between predictors**

Full Model 1 = *lmer* (Affect ~ (Population + Condition + Reward + z.Age)^2 +

z.PositioninVideoLR + z.Sex + z.CollabColor + z.trial +

(1 + Condition.I + Reward.L || Dyad) +

(1 + Condition.I + Reward.L || ID) +

(0 + z.trial | Dyad) + (0 + z.trial | ID) + (1 | trial.id) )

***Fixed Effects of Predictors***

|  | **LRT** | **df** | ***p*** |
| --- | --- | --- | --- |
| Population*Condition | 31.53 | 2 | < .001 |
| Population*Reward | 2.73 | 2 | .255 |
| Population*Age | 0.78 | 2 | .678 |
| Condition*Reward | 0.11 | 1 | .743 |
| Condition*Age | 3.87 | 1 | .049 |
| Reward*Age | 1.06 | 1 | .302 |

*no 2-way interaction between reward and other predictors, reduced model with main effect of reward is run instead

**Model 1 – Reduced Model comprising ME of reward**

Reduced Model 1 = *lmer* (Affect ~ (Population + Condition + z.Age)^2 + Reward +

z.PositioninVideoLR + z.Sex + z.CollabColor + z.Trial +

(1 + Condition.I + Reward.L || Dyad) +

(1 + Condition.I + Reward.L || ID) +

(0 + z.Trial | Dyad) + (0 + z.Trial | ID) + (1 | Trial.id) )

***Fixed Effects of Predictors***

|  | **LRT** | **df** | ***p*** |
| --- | --- | --- | --- |
| Population*Condition | 31.62 | 2 | < .001 |
| Population*Age | 0.78 | 2 | .678 |
| Condition*Age | 3.85 | 1 | .050 |
| Reward | 15.03 | 1 | < .001 |

**Step 3:**

**Model 1 – Separate Analyses per Subsample**

Full Model 1_Subsamples = *lmer* (Affect ~ Condition + z.Age + Reward +

z.PositioninVideoLR + z.Sex + z.CollabColor + z.trial +

(1 + Condition.I + Reward.L || Dyad) +

(1 + Condition.I + Reward.L || ID) +

(0 + z.trial | Dyad) + (0 + z.trial | ID) + (1 | trial.id) )

***German Subsample: Fixed Effects of Predictors***

|  | **LRT** | **df** | ***p*** |
| --- | --- | --- | --- |
| Condition | 43.96 | 1 | < .001 |
| Age | 0.01 | 1 | .069 |
| Reward | 10.39 | 1 | .001 |

**mean_High Reward_ = 1.53; mean_Low Reward_ = 1.36*

***Hai||om Subsample: Fixed Effects of Predictors***

|  | **LRT** | **df** | ***p*** |
| --- | --- | --- | --- |
| Condition | 0.85 | 1 | .358 |
| Age | 0.01 | 1 | .927 |
| Reward | 2.43 | 1 | .119 |

**mean_High Reward_ = 1.37; mean_Low Reward_ = 1.31*

***Ovambo Subsample: Fixed Effects of Predictors***

|  | **LRT** | **df** | ***p*** |
| --- | --- | --- | --- |
| Condition | 13.85 | 1 | < .001 |
| Age | 0.84 | 1 | .359 |
| Reward | 3.48 | 1 | .062 |

**mean_High Reward_ = 1.43; mean_Low Reward_ = 1.35*

**Details on Model 2**

**Step 1:**

**Comparing Full Model with a null model not comprising the predictors**

***Model Comparison***

|  | **Chisq** | **df** | ***p*** |
| --- | --- | --- | --- |
| Full Model | 26.34 | 5 | < .001 |

**Step 2:**

**Model 2 – Full Model comprising 2-way interaction between predictors**

Full Model 2= g*lmer* (FC ~ (Population + z.Age)^2 +

z.Sex +

(1 | Dyad) )

***Fixed Effects of Predictors***

|  | **LRT** | **df** | ***p*** |
| --- | --- | --- | --- |
| Population*Age | 7.29 | 2 | .026 |

**Model 2 – Exploratory Model comprising affect index as an additional predictor**

Exploratory Model 2 = g*lmer* (FC ~ (Population + z.Age + Affect Index)^2 +

z.Sex +

(1 | Dyad) )

***Fixed Effects of Predictors***

|  | **LRT** | **df** | ***p*** |
| --- | --- | --- | --- |
| Population*Age | 9.07 | 2 | .011 |
| Population*Affect Index | 1.15 | 2 | .562 |
| Age*Affect Index | 4.83 | 1 | .028 |

**Step 3:**

**Model 2 – Separate Analyses per Subsample**

Full Model 2_Subsamples = g*lmer* (FC ~ z.Age +

z.Sex +

(1 | Dyad) )

***German Subsample: Fixed Effects of Age***

|  | **LRT** | **df** | ***p*** |
| --- | --- | --- | --- |
| Age | 2.40 | 1 | .121 |

***Hai||om Subsample: Fixed Effects of Age***

|  | **LRT** | **df** | ***p*** |
| --- | --- | --- | --- |
| Age | 0.01 | 1 | .939 |

***Ovambo Subsample: Fixed Effects of Age***

|  | **LRT** | **df** | ***p*** |
| --- | --- | --- | --- |
| Age | 6.12 | 1 | .013 |

**Details on Model 3**

**Step 1:**

**Comparing Full Model with a null model not comprising the predictors**

***Model Comparison***

|  | **Chisq** | **df** | ***p*** |
| --- | --- | --- | --- |
| Full Model | 10.53 | 3 | .015 |

**Step 2:**

**Model 3 – Full Model comprising 2-way interaction between predictors**

Model 3 = lmer (ToM ~ (FC + Affect Index)^2 +

Population + z.Age + z.Sex +

(1 | Dyad) )

***Fixed Effects of Predictors:***

|  | **LRT** | **df** | ***p*** |
| --- | --- | --- | --- |
| FC*Affect Index | 0.40 | 1 | .526 |

**Model 3 – Reduced Model comprising MEs of predictors**

Model 3_Subsamples = lmer (ToM ~ FC + Affect Index +

z.Age + z.Sex +

(1 | Dyad) )

***Fixed Effects of Predictors:***

|  | **LRT** | **df** | ***p*** |
| --- | --- | --- | --- |
| FC | 3.02 | 1 | .082 |
| Affect Index | 7.61 | 1 | .006 |

**Step 3:**

**Model 3 – Separate Analyses per Subsample**

***German Subsample: Fixed Effects of Predictors***

|  | **LRT** | **df** | ***p*** |
| --- | --- | --- | --- |
| FC | 2.60 | 1 | .107 |
| Affect Index | 5.40 | 1 | .020 |

***Hai||om Subsample: Fixed Effects of Predictors***

|  | **LRT** | **df** | ***p*** |
| --- | --- | --- | --- |
| FC | 3.02 | 1 | .082 |
| Affect Index | 7.61 | 1 | .006 |

***Ovambo Subsample: Fixed Effects of Predictors***

|  | **LRT** | **df** | ***p*** |
| --- | --- | --- | --- |
| FC | 3.02 | 1 | .082 |
| Affect Index | 7.61 | 1 | .006 |
