# Supplementary material for: Cultural variation in young children’s social motivation for peer collaboration and its relation to the ontogeny of Theory of Mind
Source: PLoS One. 2020 Nov 19;15(11):e0242071. doi: 10.1371/journal.pone.0242071 (PMC7676710; doi:10.1371/journal.pone.0242071)
Supplement: S2 File — (DOCX) [file pone.0242071.s002.docx]

**– Codebook –**

**Data**

ID: [1; 2; 3; etc.] – ID for each participant

Population: [Haikom; Ovambo; Germany] – Cultural context

Age: [3.54 - 8.35] – Age of participant in decimal digits

Sex: [F; M] – Female, Male

Dyad: [O_1; H_2; etc.] – ID for each dyad

CollabColor: [Red; Blue] – Color of rope attached to collaboration device

PositioninVideoLR: [L; R] – Position of Child (Left, Right)

EE_Test1: [0 - 4] – expressed positive emotions during trial 1

EE_Test2: [0 - 4] – expressed positive emotions during trial 2

EE_Test3: [0 - 4] – expressed positive emotions during trial 3

EE_Test4: [0 - 4] – expressed positive emotions during trial 4

EE_Test5: [0 - 4] – expressed positive emotions during trial 5

EE_Test6: [0 - 4] – expressed positive emotions during trial 6

EE_Test7: [0 - 4] – expressed positive emotions during trial 7

EE_Test8: [0 - 4] – expressed positive emotions during trial 8

FC: [0;1] – forced choice behavior

(0 = individual option, 1 = collaboration)

ToM: [0 - 5] – Theory of Mind score

Affect Index: [-4 - 4] – Mean EE during collaboration trials – Mean EE during

individual trials (computed)

**Counterbalancing**

ID: [1; 2; 3; etc.] – ID for each participant

Trial: [1 - 8] – Trial in which expressed emotions are assessed

Condition: [I; C] – Condition (I = Individual; C = Collaboration)

Reward: [H; L] – Reward value of ball (H = High; L = Low)
